# Bridging the neuro-AI chasm: a framework for scalable, contextually adaptive training resources in large-scale brain data science

**DOI:** 10.3389/fpsyg.2026.1849742

**Published:** 2026-06-02

**Authors:** Mathew Abrams, Damian Okaibedi Eke, Johannes Passecker, JB Poline, John Van Horn, Milagros Marín

**Affiliations:** 1 INCF Secretariat, Karolinska Institutet, Stockholm, Sweden; 2 School of Computer Science, University of Nottingham, Nottingham, United Kingdom; 3 Institute of Systems Neuroscience, Medical University of Innsbruck, Innsbruck, Austria; 4 Department of Neurology and Neurosurgery and School of Computer Science, McGill University, Montreal, QC, Canada; 5 Department of Psychology and UVA School of Data Science, University of Virginia, Charlottesville, VA, United States; 6 DataJoint Inc., Houston, TX, United States

**Keywords:** AI in education, brain data science, cognitive offloading, desirable difficulties, metacognition, neuroinformatics, neuroscience training

## Abstract

Despite the increasing availability of large-scale brain data tools, many researchers struggle to use them effectively alongside AI. This is not due to a lack of access, but because existing training resources emphasize proficiency with these tools over critical reasoning. AI accelerates workflows but also risks deepening skill disparities: researchers with strong foundational knowledge can integrate AI-generated insights, while others become dependent on automated outputs without fully understanding their limitations, inducing risks to competency acquisition. Conventional training approaches assume that exposure to AI tools naturally translates to expertise, overlooking the need for structured cognitive engagement. We propose five cognitive science-based principles to rethink neuroscience training, ensuring AI serves as a scaffold for deeper scientific reasoning rather than a passive automation tool. We demonstrate the applicability of these principles in neuroscience education through a case study of EBRAINS training resources.

## 1 Introduction

Neuroscience is undergoing a profound transformation in how experimental data are generated, structured, shared, and analyzed. The growing scale and complexity of modern datasets—driven by advances in high-density electrophysiology, large-scale optical imaging, and whole-brain connectomics—have motivated the development of (i)unifying data standards and formats, with tools such as BIDS (Gorgolewski et al., 2016), NWB (Rübel et al., 2022), and OME-Zarr (Moore et al., 2023); (ii)public data-sharing repositories, such as DANDI Archive (2019), OpenNeuro (Markiewicz et al., 2021), and EBRAINS (Amunts et al., 2022); (iii)open-source analysis software and pipelines, such as SpikeInterface (Buccino et al., 2020), DeepLabCut (Mathis et al., 2018), CaImAn (Giovannucci et al., 2019), fMRIPrep (Esteban et al., 2019); and (iv)cloud-compatible platforms, such as BrainLife (Hayashi et al., 2024), DataJoint (Yatsenko et al., 2021), and NeuroCAAS (Abe et al., 2022). Together, these developments constitute Brain Data Science—the integrated practice of working with neuroscience data at scale across distributed repositories, multimodal pipelines, and AI-assisted workflows. However, many researchers—especially those without formal computational training—struggle to integrate these tools into their workflows in ways that are scientifically rigorous and contextually appropriate. While community-driven initiatives—from workshops and hackathons to virtual training consortia—have made significant strides in addressing this gap (Abrams and Van Horn, 2024), the need for a coherent pedagogical framework to guide the teaching of these tools remains unmet. The issue might not just be the technical complexity of these platforms but the inadequacy of training resources, which prioritize procedural fluency over conceptual understanding. Studies on digital transformation in neuroscience (Amunts et al., 2024) and in higher education more broadly indicate that many institutions lack coherent training strategies, resulting in digital skill gaps and limited faculty preparedness to integrate AI and automation effectively into research and education (Singun, 2025).

Despite rapid advances in AI-enabled neuroscience tools, current training paradigms remain primarily procedural, often emphasizing tool execution over scientific reasoning. Thus, learners may successfully operate automated workflows yet lack the conceptual frameworks required to interrogate AI-generated outputs, assess methodological assumptions, or troubleshoot unexpected results. We define this disconnect between analytical automation and human interpretive competence as the Neuro-AI chasm. While AI has the potential to enhance critical thinking when used as an interactive learning tool (Kasneci et al., 2023; Arfaie et al., 2024), neuroscience training materials often fail to incorporate it in ways that actively engage learners in reasoning and hypothesis evaluation. Instead, many resources focus on procedural automation, leaving users to accept outputs uncritically or struggle to troubleshoot errors when faced with unexpected data variations.

Similar challenges have been identified in broader digital transformation efforts in higher education, where a lack of digital literacy and misaligned training approaches contribute to ineffective adoption of AI-driven tools (Singun, 2025). Addressing this gap requires a fundamental shift in training design—one that moves beyond step-by-step procedural instruction and exposure to AI tools to actively cultivate scientific reasoning. We propose five key principles, grounded in cognitive science, to reshape neuroscience training. These principles challenge the conventional assumption that effective training relies on rigid, procedural instruction. Instead, they emphasize metacognition, productive struggle, and adaptive learning to foster deeper scientific reasoning. This framework ensures AI functions as a collaborator that enhances—rather than replaces—critical engagement with neuroscientific data.

Whilst this paper articulates specifically the “AI Paradox” (see also West et al., 2024, for a separate but detailed discussion) our proposed five cognitive science-based principles for training design apply to many types of neuroscience training modules involving AI models. We demonstrate their application through EBRAINS training resources. We also discuss how this framework complements evaluation tools such as NERC and open platforms such as INCF Training Space. While these cognitive foundations are recognized across multiple scientific education contexts—including bioinformatics training, computational biology pedagogy, and physics education research—the present framework is specifically tailored to neuroscience training through the discipline-specific applications in Section 4, the case study in Section 5, and the FAIR comparison in Section 6.2.

## 2 Background: the training gap in AI-assisted neuroscience

### 2.1 The AI paradox

AI has reshaped neuroscience training, lowering technical barriers while posing significant risks for genuine competency acquisition. This contrast—what we term the AI Paradox—captures AI’s dual role: enhancing the availability of knowledge while increasing the risk of cognitive offloading, in which users rely on automation without deeply engaging with the underlying science (Dubois, 2024). While AI-powered tools streamline workflows, they also introduce automation bias—the tendency to accept algorithmic outputs uncritically rather than questioning their validity (Gerlich, 2025).

Studies on AI-assisted learning in higher education suggest that many training programs emphasize procedural tool use over conceptual understanding, thereby reinforcing reliance on automation at the expense of analytical reasoning (Singun, 2025; Gkintoni et al., 2025). This approach, while improving technical fluency, often fails to develop the critical thinking skills necessary to interrogate AI-generated findings. As a result, researchers with strong foundational knowledge benefit most, while others risk becoming overly dependent on automation (Gerlich, 2025), potentially deepening disparities in expertise rather than closing them.

### 2.2 The simplification-abstraction trap

Training materials frequently oversimplify complex neuroscientific methods to increase accessibility, often at the cost of conceptual depth. AI-powered tools exacerbate this tendency by automating complex analytical steps, making it tempting for training designers to abstract away the very processes learners need to understand. While simplification aids initial engagement, excessive content reduction can hinder the development of critical thinking and problem-solving skills necessary for real-world applications (Gkintoni et al., 2025). Neuroscience training must ensure that learners develop a solid conceptual framework to transition from guided exercises to independent research.

### 2.3 AI hallucinations and misinformation risks

AI-assisted content, even when not fully AI-generated, can introduce biases, factual inaccuracies, and fabricated references, undermining trust in training materials (Dubois, 2024). These distortions may stem from biases embedded in training datasets, AI’s misinterpretation of incomplete data, or users’ uncritical acceptance of AI-generated insights. Ethical, privacy, and regulatory concerns regarding AI-generated educational content add further complexity that training programs often overlook (Dubois, 2024). Neuroscientists require not only proficiency in AI-assisted analysis but also the ability to critically evaluate AI outputs, assess reliability, and recognize potential biases in algorithmic decision-making. For example, LLMs frequently fabricate citations—a risk learners must be explicitly trained to recognize.

## 3 Methods: framework development

### 3.1 Approach

The five principles presented in this paper emerged from an iterative process integrating three sources: (1) a review of cognitive science literature on learning and skill acquisition, (2) analysis of existing neuroscience training resources and their limitations, and (3) structured discussions within the Global Alliance for Genomics and Health (GA4GH) and INCF Neuroscience Community Scientific Collaboration and Education Theme Team.

The cognitive science foundation’s draw primarily on research regarding desirable difficulties in learning (Bjork, 1994; Bjork and Bjork, 2020), metacognitive monitoring (Fan et al., 2025), and collaborative problem-solving (Haduong et al., 2024). These principles were then contextualized for computational neuroscience education, where learners must simultaneously develop competencies in programming, data analysis, mathematical modeling, and neurobiological interpretation.

### 3.2 Principle selection criteria

Principles were selected for their grounding in peer-reviewed cognitive science, specific relevance to AI-induced learning risks, and practical implementability in existing training formats.

## 4 Five principles for brain data training resources

The five principles operate at complementary cognitive and social levels (Table 1). Together, they form an integrated framework designed to counteract AI-induced cognitive offloading while preserving the efficiency benefits of automation; they do not function sequentially but interact dynamically within training environments.

**Table 1:** Five principles for brain data training resources: cognitive foundations, AI-specific risks addressed, and practical applications.

Principle	Cognitive foundation	AI risk addressed	Neuroscience application
1. Productive struggle	Desirable difficulties enhance long-term retention (Bjork, 1994; Bjork and Bjork, 2020)	Automation removes beneficial challenge, creating passive learners	Spike sorting with raw data before comparing with automated results
2. Contextualized case studies	Ecological validity: learning must connect to learners’ real working conditions	AI tools assume uniform infrastructure, excluding resource-constrained settings (see Schirner et al., 2022)	Calcium imaging workflows with cloud and local hardware tiers (Beltran-Parrazal et al., 2014)
3. Adaptive learning	Scaffolding calibrated to expertise prevents overload and passivity (Gkintoni et al., 2025)	One-size-fits-all tutorials fail diverse learners	Branching DataJoint/NWB tutorials with fading AI hints
4. Metacognitive reflection	Metacognitive monitoring prevents the illusion of competence (Fan et al., 2025)	AI outputs accepted without scrutiny	Reflection checkpoints after automated functional magnetic resonance imaging (fMRI) preprocessing
5. Collaborative problem-solving	Collaborative cognition can outperform individual and automated approaches (Haduong et al., 2024)	AI replaces discourse with automated answers	Multi-modal integration treating AI as a “provocateur” rather than an oracle

### 4.1 Principle 1: design for neuro-specific productive struggle

*Rationale*: Learning is most effective when it involves “desirable difficulties”—challenges that enhance long-term retention and comprehension by engaging encoding and retrieval processes (Bjork, 1994). Neuroscience training should integrate tasks that balance challenge and support to develop deeper conceptual understanding and problem-solving skills (Bjork and Bjork, 2020).

*Implication*: Although rigid, guided tutorials may have their place in learning such things as code syntax, subsequent training should shift to inquiry-driven modules that use complex, real-world datasets. Key strategies include spacing practice, interleaving topics, retrieval practice through self-testing, encouraging learners to generate rather than consume answers, calibrating task difficulty, and framing errors as learning opportunities (Bjork and Bjork, 2020).

*Computational neuroscience application:* In spike-sorting tutorials, rather than providing pre-sorted data, present learners with raw electrophysiological recordings that contain ambiguous cases. Require them to make sorting decisions, justify their reasoning, and compare their results with automated algorithms; thus, enabling learners to experience productive struggle that builds genuine expertise. A tutorial should provide a gold-standard comparison after the student completes the task to solidify the learning from errors strategy.

### 4.2 Principle 2: contextualize case studies for local resource environments

*Rationale*: While core scientific methods must strive to be universal, their practical application depends on local resource constraints and infrastructural realities.

*Implication*: Develop adaptable case studies which instructors can customize to reflect local challenges, such as limited access to high-end equipment, ensuring that the teaching materials remain relevant and effective without altering the universal scientific principles.

*Computational Neuroscience Application:* A calcium imaging analysis tutorial (for instance, see Beltran-Parrazal et al., 2014; Gerasimov et al., 2023) should provide workflows with scalable tiers of complexity—where modern examples might be provided with some utilizing cloud GPU resources for large-scale processing (Dong et al., 2021; Hayashi et al., 2024) and others optimized for local computation on standard hardware (Kim et al., 2023). This ensures learners in resource-constrained environments can engage with the same scientific concepts without being excluded by infrastructure requirements.

### 4.3 Principle 3: redesign tutorials for adaptive learning

*Rationale*: Many educational materials in neuroscience rely on rigid, step-by-step tutorials that prioritize procedural efficiency over conceptual understanding. While AI-driven systems can provide individualized guidance, current training resources fail to leverage AI as a tool for fostering deeper learning. Standardized tutorials often present information in a static, linear fashion, which benefits advanced learners but overwhelms novices who require gradual scaffolding (Gkintoni et al., 2025).

*Implication*: Rather than relying on fixed tutorials, educators should implement flexible, inquiry-driven training modules that dynamically adjust to different levels of expertise and encourage independent problem-solving.

*Computational neuroscience application*: A DataJoint or NWB tutorial could implement branching pathways: novices receive additional scaffolding on database concepts before tackling neuroscience-specific schemas, while experienced programmers proceed directly to advanced query optimization. Both pathways converge on the same learning objectives but respect different starting points. As the learner progresses through the DataJoint/NWB tutorial, the AI or tutorial hints should gradually disappear, forcing the learner to rely on the conceptual framework developed in Principle 1 (Design for Neuro-Specific Productive Struggle).

### 4.4 Principle 4: embed metacognitive reflection

*Rationale*: Current training resources fail to develop learners’ ability to critically evaluate AI-generated outputs, leading to overreliance and misinterpretation. Automated analyses (e.g., spike sorting) are often accepted without scrutiny, reinforcing an illusion of competence and reducing self-regulated learning (Fan et al., 2025).

*Implication*: Embed systematic reflection checkpoints and interactive modules that require learners to compare AI-generated outputs with human-generated alternatives, assessing differences in reasoning, accuracy, and reliability. This practice strengthens critical thinking by actively engaging learners in identifying errors and understanding the limitations of AI-assisted analysis (e.g., by comparing automated and manual spike-sorting accuracy). Additionally, instructors should intentionally design AI-assisted tasks to complement—not replace—core analytical and problem-solving processes. Scaffolding strategies should be implemented to stimulate intrinsic motivation, reinforce self-directed learning, and promote AI literacy, ensuring that AI serves as an aid rather than a substitute for critical evaluation (Fan et al., 2025).

*Computational Neuroscience Application:* After running an automated preprocessing pipeline on fMRI data, require learners to answer: “What assumptions did this pipeline make? Which parameters would you change for a different population? What artifacts might it have missed?” This metacognitive pause transforms passive execution into active scientific reasoning.

### 4.5 Principle 5: Foster collaborative problem-solving in multi-modal analysis

*Rationale*: AI-driven workflows prioritize efficiency but often overlook the value of collaborative scientific discourse and debate. Research indicates that collaborative problem-solving —whether in human-only teams or human-AI partnerships—can outperform both individual and fully automated approaches in resolving complex, ambiguous scientific questions (Haduong et al., 2024). In neuroscience, where multi-modal data integration requires interpretation across diverse datasets, collaboration enhances error detection, hypothesis refinement, and methodological innovation.

*Implementation*: AI should not function as a passive analysis tool but as a dynamic contributor to scientific inquiry. To capture this shift, we adopt the framing of AI as a “provocateur”: an interlocutor whose role is not to deliver authoritative answers but to surface alternative hypotheses, expose unstated assumptions, and prompt iterative scientific debate. Reframing AI in this way directly counteracts the cognitive offloading and automation bias described in Section 2.1 (The AI Paradox) and operationalizes Principle 5 in a way that complements (rather than replaces) human reasoning. In practice, the “provocateur” role can be implemented through several complementary mechanisms:

- AI-driven challenges to working assumptions, prompting peer discussion and iterative refinement of conclusions.
- Integrate adversarial pipelines—Design AI workflows that simulate contradictory analyses, requiring researchers to evaluate inconsistencies and develop stronger scientific reasoning (e.g., contrasting electrophysiology preprocessing strategies).
- Encourage structured team-based learning—Develop educational exercises in which learners engage in AI-assisted group analysis, comparing human-led and AI-driven insights while negotiating methodological decisions.
- Embed transparency checkpoints—AI tools should be designed with built-in transparency mechanisms, ensuring that results are not blindly accepted but instead contextualized through collaborative verification.

*Computational neuroscience application:* In a multi-modal integration exercise combining electrophysiological and imaging data, have the teams use different AI-assisted analysis approaches, then present and defend their findings to peers. The AI serves as a “collaborator,” generating hypotheses that humans must critically evaluate, not as an oracle delivering final answers.

## 5 Case study: applying the five principles to EBRAINS BrainScaleS-2 training resources

To demonstrate the practical applicability of the five principles, we examined their implementation within the BrainScaleS-2 ecosystem (Pehle et al., 2022; BrainScaleS-2 Documentation, 2023), a specific knowledge and training resource associated with the *EBRAINS Research and Education Infrastructure*. BrainScaleS-2 is an advanced neuromorphic hardware platform that physically mimics the biological behavior of neurons and synapses. The ecosystem also serves as a substantial pedagogical training resource for different educational levels (high-school to university students) by making complex neuroscientific and computational concepts tangible. The platform primarily utilizes documentation and interactive Jupyter notebooks with Python-based APIs, allowing users to explore brain functions without deep coding knowledge. Conversely, advanced notebooks challenge university students with complex programming and physics-based laboratory tasks using PyNN (Davison et al., 2009).

To fully utilize the BrainScaleS-2 training ecosystem, users must register for an EBRAINS account. While this creates a minor barrier for individuals, it critically enables access to high-performance computing infrastructure that is rarely available to educators globally, aligning with Principle 2 (Contextualize Case Studies for Local Resource Environments). Overall, the ecosystem provides a structured, iterative learning flow with increasing difficulty, from introductory Python usage to modeling advanced spiking neural networks. The training resource further integrates Lu.i (Stradmann et al., 2025), an affordable and accessible physical electronic neuron circuit that illustrates the basic dynamics of biological neurons. This hands-on approach motivates students to engage in active learning and self-testing, which aligns directly with Principle 1 (Design for Neuro-Specific Productive Struggle).

Furthermore, most exercises and tutorials include questions that encourage students to explore alternatives, devise solutions to unseen problems, and reflect on the tutorial’s underlying assumptions (Principle 4: Embed Metacognitive Reflection). The deployment of these resources could be further expanded in line with Principle 5 (Foster Collaborative Problem-Solving in Multi-Modal Analysis) through international mentored programs (e.g., Google Summer of Code) or local group-work settings where adversarial teams or AI agents challenge assumptions and present alternative methodologies.

Despite these strengths, our analysis identifies a specific opportunity regarding Principle 3 (Redesign Tutorials for Adaptive Learning). Future tutorials should integrate additional checkpoints and branching paths that require learners to develop and explore alternatives (potentially with AI assistance) to successfully complete exercises. While the BrainScaleS-2 ecosystem does not currently integrate such AI-assisted learning, its technical richness makes it an ideal testbed for developing these proposed principles into a fully integrated learning environment.

## 6 Discussion

### 6.1 Tensions in neuro-AI training design

A central tension in AI training concerns the balance between throughput and translucency. AI systems can dramatically accelerate technical workflows and lower barriers to entry for increasingly complex computational methods. Tasks such as calcium imaging analysis, electrophysiological preprocessing, spike sorting, or large-scale neuroimaging workflows that once required extensive manual effort can now be performed rapidly with AI-assisted pipelines (see, for example Juneau et al., 2026). While this acceleration improves efficiency and broadens participation, it also introduces the risk that learners may engage with these tools as opaque systems rather than as interpretable scientific instruments. Critical conceptual and methodological steps, including artifact detection, quality control, parameter selection, and assumptions embedded within preprocessing pipelines, may become hidden beneath layers of automation. As a result, learners may acquire procedural fluency without developing the deeper conceptual understanding necessary to critically evaluate outputs, identify failure modes, or recognize biologically implausible results. Effective Neuro-AI education must therefore balance efficiency with transparency, ensuring that AI-assisted workflows remain pedagogically interpretable rather than functioning as inaccessible “black boxes.”

A related challenge concerns infrastructure equity in neuroscience education. Many advanced AI-driven neuroscience methods assume access to substantial computational resources, including high-performance GPUs, cloud infrastructure, and specialized software environments. Such assumptions risk exacerbating existing disparities between well-resourced institutions and those with more limited technical capacity. Training frameworks must therefore be adaptable across a broad spectrum of educational and research settings. In practice, this means emphasizing scalable pedagogical strategies that preserve conceptual learning even when sophisticated infrastructure is unavailable. Lightweight computational approaches, browser-based educational tools, smartphone-enabled imaging technologies, and cloud-accessible demonstrations can provide meaningful engagement with modern neuroscience methods while reducing dependence on expensive local hardware. By designing educational resources that remain effective across diverse environments, Neuro-AI training can become more globally accessible and inclusive.

Beyond these cross-cutting tensions, the five principles vary in implementation effort. Principles 1 and 4 (Productive Struggle and Metacognitive Reflection) can typically be incorporated into existing tutorials with modest curricular adjustments—for example, adding reflection prompts or replacing pre-sorted data with raw recordings. In contrast, Principle 3 (Adaptive Learning) and Principle 5 (Collaborative Problem-Solving) demand substantially greater design effort, requiring branching pathways, multi-modal exercises, and in some cases AI-assisted scaffolding infrastructure. Principle 2 (Contextualized Case Studies) falls between these extremes. Given that educators often lack institutional support, time, and resources to redesign training materials, we recommend prioritizing the lower-cost principles as entry points, while pursuing the higher-cost principles incrementally through funded development efforts and community collaborations.

### 6.2 Differentiation from FAIR principles

Importantly, this framework differs in emphasis from existing standards such as the FAIR principles (Wilkinson et al., 2016). FAIR has fundamentally reshaped neuroscience by promoting robust data sharing, standardization, and reproducibility (Abrams et al., 2021). However, FAIR primarily addresses the organization and accessibility of scientific resources rather than the cognitive and pedagogical processes through which researchers learn to reason with those resources. Our framework instead focuses on the educational dimensions of Neuro-AI integration: how learners develop conceptual understanding, critical thinking, and adaptive reasoning skills while interacting with increasingly automated systems. Rather than emphasizing only the standardization of datasets and workflows, this approach prioritizes the cultivation of scientific judgment. Learners must be trained not simply to consume AI-generated outputs, but to interrogate them critically, contextualize them biologically, and understand the assumptions and limitations underlying algorithmic decisions. In this sense, the framework complements FAIR principles by extending attention from logistical interoperability to human-centered learning, collaborative reasoning, and pedagogical adaptability across diverse resource contexts (see also the work of Reer et al., 2023).

### 6.3 Limitations

Several limitations of the present framework should also be acknowledged. First, the proposed principles emerge from theoretical synthesis, cognitive science literature, and expert discussion rather than from systematic empirical validation within neuroscience training environments. Although the principles are grounded in established educational and cognitive theory, their effectiveness in computational neuroscience and Neuro-AI education remains to be evaluated through longitudinal and comparative study designs. Additional empirical work will be necessary to determine how these principles influence learner comprehension, retention, research independence, and long-term methodological competence.

Second, while this framework emphasizes the potential risks associated with AI-driven cognitive offloading, automation itself is not inherently detrimental to learning. In many contexts, well-designed AI systems may reduce unnecessary cognitive burden and allow learners to devote greater attention to higher-order reasoning, hypothesis generation, and scientific interpretation. The challenge is therefore not whether automation should be incorporated, but rather how it should be integrated in ways that preserve conceptual engagement. The optimal balance between manual interaction and automated assistance likely depends on learner expertise, disciplinary context, and instructional goals, suggesting that Neuro-AI educational strategies may need to evolve dynamically across stages of training.

Finally, the case study presented in Section 5 should be viewed as an initial demonstration of the framework rather than definitive validation of its broader applicability. Additional work will be required to assess the framework across multiple neuroscience subdomains, diverse learner populations, and varied educational settings. Future efforts should incorporate systematic educator feedback regarding feasibility and implementation challenges, alongside longitudinal evaluation of learner outcomes and collaborative performance. Related initiatives are also beginning to address the broader question of determining when digital neuroscience tools and resources are sufficiently mature and pedagogically reliable for educational adoption. Together, these future directions will help establish a stronger evidence base for the responsible integration of AI into neuroscience education and training.

## 7 Recommendations & Call to action

Based on the five principles and the gaps identified through the case study, we offer targeted recommendations for three key audiences (Table 2).

**Table 2:** Recommendations for bridging the Neuro-AI chasm by audience.

Audience	Recommendation	Description	Example
Educators	Teach “AI-assisted skepticism” as a science-based critical thinking best practice.	Training programs must move beyond procedural fluency, incorporating structured problem-solving exercises that cultivate neuroscientific intuition and adaptive AI guidance that differentiates support based on learners’ expertise levels.	“Use the LLM to generate and critique a data analysis plan for patch-clamp experiments.”; “Prompt engineering to surface a tool’s limitations.”
Educators	Provide for both Guest vs. power user modes for tool usage	Restrict AI features for novices until mastery milestones.	No code automation for beginners.
Developers	Develop “AI Audit Trails” as a means to track AI usage or performance	Use AI to trace decisions and error logs, providing transparent, traceable records. Analysis tools paired with LLM-generated logs to trace AI decisions.	“Why did the AI exclude this electrophysiology channel?”
Developers	Track AI-related “Depth Metrics” which measure learner mastery and engagement	Track learner progression and engagement depth.	How many users modify the provided code vs. blindly rerunning it?
Developers	Provide neuroinformatics customization modules	Develop templates that enable researchers to replace default datasets, demonstrating the adaptability of analytical methods across experimental models and thereby enhancing the relevance and applicability of training materials.	Electrophysiological data from mouse brains vs. brain organoids.
Developers	Integrate and align tools with NERC recommendations	When developing training materials, use the Neuroscience Education Readiness Checklist (NERC) to identify barriers to adoption, then apply the five principles for brain data training resources to design pedagogically sound content that addresses them.	Assess an EEG preprocessing tutorial with NERC, then apply the five principles to address identified barriers.
Societies and funding bodies	Certify training resources meeting Neuro-FAIR standards	Adapted from the FAIR data principles to address domain-specific requirements of neuroscience training resources. Benchmarks for pipeline performance across diverse populations.	“Does this spike-sorting algorithm work consistently across different neuronal firing patterns (e.g., cortical vs. subcortical recordings)?”
Societies and funding bodies	Fund global mentor networks (groups of experienced neuroscientists, educators, and AI specialists)	Stress-test tutorials across diverse neuro-ecologies, i.e., different research environments that vary in technological access, educational backgrounds, or institutional constraints.	Deploying neuroscience software training in labs with limited access to high-performance computing or specialized equipment (limited HPC access).
Societies and funding bodies	Support integrated neuroscience tool training frameworks	Support the development and validation of integrated frameworks that address both tool evaluation and training design, recognizing that effective neuroscience education requires attention to both dimensions.	Combine tool evaluation (NERC) with training design (five principles) for comprehensive neuroscience education.

## 8 Conclusion

By treating AI as a *collaborative “provocateur”*—not a replacement for human expertise—we can build training ecosystems that empower all neuroscientists to engage critically with the convergence of data standards, sharing repositories, analysis software, and cloud-compatible platforms reshaping the field.

The Neuro-AI chasm reflects not a failure of technology, nor a failure of educators—who often lack institutional support, time, and resources to redesign training materials—but rather a gap in pedagogical frameworks suited to the AI era. Most existing training approaches were developed before AI tools became ubiquitous, leaving educators to adapt without clear guidance. As AI capabilities continue to advance, the risk grows that neuroscience training will produce technicians proficient at running automated pipelines but lacking the scientific reasoning to evaluate, troubleshoot, and innovate beyond them. The five principles presented here—productive struggle, contextualized case studies, adaptive learning, metacognitive reflection, and collaborative problem-solving—offer a cognitive science-grounded approach to ensuring that AI enhances, rather than replaces, critical thinking. These principles are intended not as criticism of current practice but as practical tools to support educators navigating an unprecedented transition.

We invite the neuroscience education community—developers, educators, and scientific societies—to adopt, test, and refine this framework. Open platforms such as INCF Training Space (2019) provide existing infrastructure for hosting and disseminating training resources designed according to these principles. This effort can be used alongside tool evaluation approaches to ensure that neuroscience education keeps pace with rapid technological change. The goal is not to resist AI integration but to harness it thoughtfully, ensuring that the next generation of neuroscientists possesses both the technical fluency to leverage AI tools and the scientific reasoning to know when to question them.

## Data Availability

The original contributions presented in the study are included in the article/supplementary material, further inquiries can be directed to the corresponding author/s.
